# Plant-derived essential oil mixture from *Salvia rosmarinus, Lavandula dentata*, and *Cymbopogon citratus* a promising biofungicide against postharvest green mold *Penicillium digitatum*

**DOI:** 10.3389/fpls.2026.1927657

**Published:** 2026-08-14

**Authors:** Salahddine Chafiki, Abdallah Oukarroum, Redouan Qessaoui, Soumaya El Assri, Hasnaa Lahchimi, Mohamed Alouani, Hicham El Arroussi, Rachid Bouharroud

**Affiliations:** 1 Regional Center of Agricultural Research of Agadir, National Institute of Agricultural Research (INRA), Rabat, Morocco; 2 AgroBioSciences Department (AgBS), Mohammed VI Polytechnic University (UM6P), Ben Guerir, Morocco; 3 Research Team in Science and Technology, Higher School of Technology, Ibn Zohr University, Laayoune, Morocco; 4 Algal Biotechnology Center, AgroBiosciences, College of Agriculture and Environmental Sciences, Mohammed VI Polytechnic University (UM6P), Ben Guerir, Morocco; 5 Laboratory of Biotechnology and Valorization of Natural Resources, Faculty of Science, Ibn Zohr University, Agadir, Morocco; 6 Faculty of Applied Science, Ait Melloul, Ibn Zohr University, Agadir, Morocco

**Keywords:** antifungal activity, *C. citratus*, essential oil, *L. dentata*, *P. digitatum*, *S. rosmarinus*

## Abstract

Postharvest green mold, caused by *Penicillium digitatum*, is considered one of the most economically significant diseases affecting citrus fruit. Current management strategies for green mold rely primarily on the pre- and postharvest application of synthetic fungicides. Plant-derived essential oils (EOs) have been presented as sustainable and ecofriendly alternatives to synthetic pesticides. The objective of the present study is to evaluate the antifungal property of *Salvia rosmarinus, Lavandula dentata*, and *Cymbopogon citratus* EO and their mixture against *P. digitatum.* Under *in vitro* environment, all treatments exhibited a significant inhibitory effect on *P. digitatum* mycelial growth. The ternary EOs mixture exhibited significant effect compared to their individual application, showing lowest value of IC_50_ (0.0452 µL/mL). Moreover, the application of this mixture on infected fruits showed significant inhibitory effect compared to the control. The lesion diameter measured for untreated fruits was 64.29 mm, and 36.05 mm for fruits treated with EOs. In addition, disease incidence of *P. digitatum* was complete 100% in all fruits tested. Nonetheless, a significant decrease in disease severity was observed among fruits treated with EOs (56.67%). The findings of this study indicate that combining these EOs represents a promising natural strategy for controlling *P. digitatum* infections.

## 1 Introduction

*Penicillium digitatum*, the causal agent of citrus green mold (Saccardo, 1881). This species belongs to Trichocomaceae family in the class of Eurotiomycetes. Green mold ranks among the most economically damaging postharvest diseases affecting citrus fruit across all production regions characterized by low summer rainfall. This pathogen behaves as a strict wound pathogen, capable of infecting all citrus species and cultivars at multiple stages, including in the field, at packing houses, and throughout distribution and marketing channels (CABI, 2021). The pathogen initiates infection exclusively through mechanical injuries to the rind of mature citrus fruits, such as oranges, mandarins, and lemons, typically sustained during harvesting, handling, or packing operations. The economic impact of this fungus is substantial, with postharvest losses reportedly reaching up to 90% under certain conditions (Zhu et al., 2017; Lin et al., 2019). Furthermore, *P. digitatum* proliferates rapidly on fruit surfaces, and its spores are widely distributed throughout the atmosphere (Kanetis et al., 2007). Current management strategies for green mold rely primarily on the pre- and postharvest application of synthetic fungicides, including imazalil, thiabendazole, pyrimethanil, and fludioxonil (Kaplan and Dave, 1979; Smilanick et al., 2006; Kanetis et al., 2007). Nonetheless, using chemical treatments is increasingly being restricted in various countries, prompting the exploration of alternative control methods, such as plant-derived EOs.

EOs of several plants are composed of different molecules such as terpenes, terpenoids, phenylpropenes, and others that exhibit antimicrobial effects through various mechanisms. Several plants have been applied against *P. digitatum* and showed promising results (Vitoratos et al., 2013; Zulu et al., 2023; Souza et al., 2025; Haddou et al., 2026). In a study, the mixture of oregano-rosemary exhibited significant growth reduction against *P. digitatum* (Khaled et al., 2025). Likewise, clove, thyme, and Eucalyptus citriodora EO’s totally inhibited the fungus at concentrations ≤ 2 µL/mL, by both contact and volatile effect (Souza et al., 2025). Although research on EOs and their antifungal effects are wildly documented, there are knowledge gaps regarding the effect of EOs mixture, especially *in vivo* tests. The present work aims to evaluate the antifungal potential of individual and ternary EOs mixture from three plants; *L. dentata*, *S. rosmarinus*, and *C. citratus* to manage *P. digitatum in vitro* and *vivo*, with a broader goal of providing basis to eco-friendly alternatives and to contribute to sustainable disease control.

## 2 Materials and methods

### 2.1 Extraction of EO

The plant material was harvested (at the flowering stage) from the experimental farm of the National Institute of Agricultural Research’s (INRA), Morocco. Identification of plants was performed by specialists from INRA and the university Ibn Zohr. EOs were extracted from air-dried leaves of plants by hydro-distillation (3 h) using a Clevenger-type apparatus, then dried over anhydrous sodium sulfate and stored in sealed vials at 4 °C until use (Soltanbeigi and Maral, 2025).

### 2.2 Characterization of EOs

The chemical composition analysis of the EOs was previously determined by GC-MS and reported in our earlier work (Chafiki et al., 2025). The samples were analyzed using a GCMS-TQ8040 system (Shimadzu, Japan) equipped with an Rtx^®^-5MS capillary column (30 m × 0.25 mm i.d., 0.25 µm film thickness; Restek, PA, USA). Prior to injection, samples were diluted in hexane (1:10, v/v) and filtered through a 0.45 µm syringe filter. A 1 µL aliquot of each diluted sample was introduced in split injection mode, with the injector maintained at 250 °C. Helium served as the carrier gas at a constant flow rate of 1.5 mL/min. The oven temperature program began at 50 °C (held for 2 min), then increased at a rate of 5 °C/min up to 300 °C, where it was held isothermally for an additional 3 min. Mass spectrometric detection was performed under electron ionization (70 eV), with the ion source and interface held at 200 °C and 280 °C, respectively, and mass spectra recorded over a scan range of 50–500 m/z. Identification of individual compounds was based on n-alkane standards and NIST 2017 mass spectral library. For the purpose of the present study, only constituents with a relative abundance exceeding 1% of the total composition were presented.

### 2.3 *P. digitatum* isolation

The *P. digitatum* strain used in this study was isolated from a decaying orange and identified at the Laboratory of Plant Protection at INRA, Agadir, Morocco. The fungal culture was maintained on potato dextrose agar (PDA) at 28 ± 2 °C until sporulation occurred. The fungus was purified by single-spore isolation and subcultured onto fresh PDA plates to obtain a pure culture (Qessaoui et al., 2022). Identification of the isolate was carried out on the basis of macroscopic and microscopic morphological criteria, following the taxonomic keys of Pitt and Hocking (2022). The plates were subsequently stored at 4 °C. The pathogenicity of the isolated strain was confirmed by artificial inoculation on healthy orange fruits, followed by re-isolation of the fungus from the resulting lesions.

### 2.4 Determination of the *in vitro* antifungal potential of EOs

#### 2.4.1 Volatile activity assay

The antifungal potential of the three individual EOs and their combination against *P. digitatum* mycelial growth was assessed through the volatile activity (VA) assay, following the procedure outlined by Chafiki et al. (2025). Briefly, a single mycelial disc of 5 mm was deposited at the center of sterile Petri dishes containing PDA. Sterile discs (25 mm) of filter paper (Whatman 1) were piped with the EOs at five concentrations ranging from 0.02 to 0.32 µL/mL of Petri dish air volume. These treated discs were fixed to the inner side of the Petri dish lids. After 7 days of incubation at 24 ± 1 °C, the mycelial growth inhibition (MGI) rates were then determined according to the formula reported by Pandey et al. (1982):


$$MGI\left(\%\right)=\frac{\text{Mycelial diameter in control}-\text{Mycelial diameter with treatement}}{\text{Mycelial diameter in control}}\times 100$$

The assay was performed in triplicate (n=3) and repeated twice.

#### 2.4.2 Direct contact test

Based on the results of volatile assay, the mixture of EOs at ratio of (1:1:1v/v/v) showed significant mycelial growth inhibition of *P. digitatum* compared to their individual application. This treatment was selected for this test. Antifungal effect of this mixture was assessed by incorporating the EOs mixture dissolved in Tween 80 (0.1%) into PDA medium. The concentrations tested ranged from 0.2 to 3.2 μL/mL. The negative control was PDA containing 0.1% Tween 80. Each Petri dish was inoculated with 5 mm of fungus. After 7 days of incubation at 24 ± 1 °C, the antifungal effect was evaluated in terms of mycelial growth inhibition percentage (Chafiki et al., 2025). The assay was performed in triplicate (n=3) and repeated twice.

#### 2.4.3 Spores’ germination test

A suspension of *P. digitatum* (10^6^ spores/mL) was combined with Sabouraud dextrose broth containing the EO mixture at various concentrations ranged from 0.2 to 3.2 µL/mL, mixed at a 1:1 (volume) ratio. An equivalent volume of spore suspension with Sabouraud dextrose broth was used for control. After 24 h of incubation at 24 ± 1 °C, the spores’ germination was subsequently examined using a microscope, with at least 100 spores assessed per replicate. The spore was considered germinated once the length of germ tube exceeded the spore’s largest diameter. The percentage inhibition of spore germination (IG) was then calculated using the following equation (Soylu et al., 2010):


$$\text{IG}\left(\%\right)=\frac{\left(\text{Number of spores germinated in controls}-\text{Number of spores germinated with treatment}\right)}{\text{Number of spores germinated in controls}}\times 100$$

The assay was performed in triplicate (n=3) and repeated twice.

### 2.5 *In vivo* antifungal activity of EOs mixture

Untreated Citrus sinensis fruits used in the antifungal assay were sourced from the INRA experimental farm (Melk Zhar) in Belfaa, Morocco (30°02′42.2″N, 9°33′13.4″W). Fruit selection was based on healthy appearance, uniform size, and the absence of bruising or visible disease symptoms. Prior to wounding, fruits were surface disinfected by immersion in sodium hypochlorite solution (1%) for 2 min, followed by rinsing with distilled water and air-drying under a laminar flow hood. A standardized wound (2 mm deep, 4 mm in diameter) was then made on the equatorial region of each fruit. Each wound was piped with 40 µL of the EO mixture at the IC_90_ concentration dissolved in Tween 80 (0.1%), followed 30 min later by inoculation with 20 µL of a *P. digitatum* suspension (10^6^ spores/mL). Control samples were pipetted with 40 μL of sterile Tween 80 (0.1%). Inoculated fruits were placed in plastic containers and incubated at 25 °C. Lesion diameters were recorded on day 5 post-inoculation, and all experiments were performed in triplicate.

Disease severity was calculated by evaluating the disease development of green mold using scale 0–4 as described in **Table 1**. The disease severity was calculated using the following formula (Chen et al., 2019; Khaled et al., 2025):

**Table 1:** Rating scale of disease severity.

Scale	Lesion diameter (LD)
0	No symptoms development
1	1 ≤ LD≤ 20 mm
2	20< LD ≤ 40 mm
3	40< LD ≤ 60 mm
4	LD > 60 mm


$$\text{Severity}\left(\%\right)=\frac{\sum \text{Disease scale}\times \text{number of fruit in each scale }}{\text{highest scale}\times \text{total number of fruit}}\times 100$$

### 2.6 Statistical analysis

Statistical analyses were carried out using RStudio (Posit Software, PBC, version 2026.04.0). Mean comparisons were assessed through the Kruskal-Wallis and Mann-Whitney U tests, with significance set at p< 0.05. In addition, OriginPro 2026 (SR1 10.3.0.197) was employed for data visualization and for determining the inhibitory concentrations of the EOs.

## 3 Results

### 3.1 GC-MS analysis of the EOs

EOs yields ranged from 0.91% to 1.02% across the three species, with *S. rosmarinus* producing 0.97 ± 0.06%, *L. dentata* 0.91 ± 0.04%, and *C. citratus* 1.02 ± 0.02% following hydrodistillation. As previously reported in our study (Chafiki et al., 2025), the chemical composition of the investigated EOs was fully characterized by GC-MS analysis, with all detected constituents being identified. Regarding the major constituents identified, *C. citratus* EO was chiefly dominated by geranial (42.91%), neral (34.11%), and β-pinene (9.32%), while *L. dentata* EO was primarily composed of camphor (33.95%), 1,8-cineole (32.35%), and β-pinene (5.23%). Meanwhile, *S. rosmarinus* EO was characterized mainly by camphor (17.60%), α-pinene (14.39%), and 1,8-cineole (14.13%).

### 3.2 *In vitro* antifungal potential of EOs on *P. digitatum*

#### 3.2.1 Volatile assay

**Figure 1:**
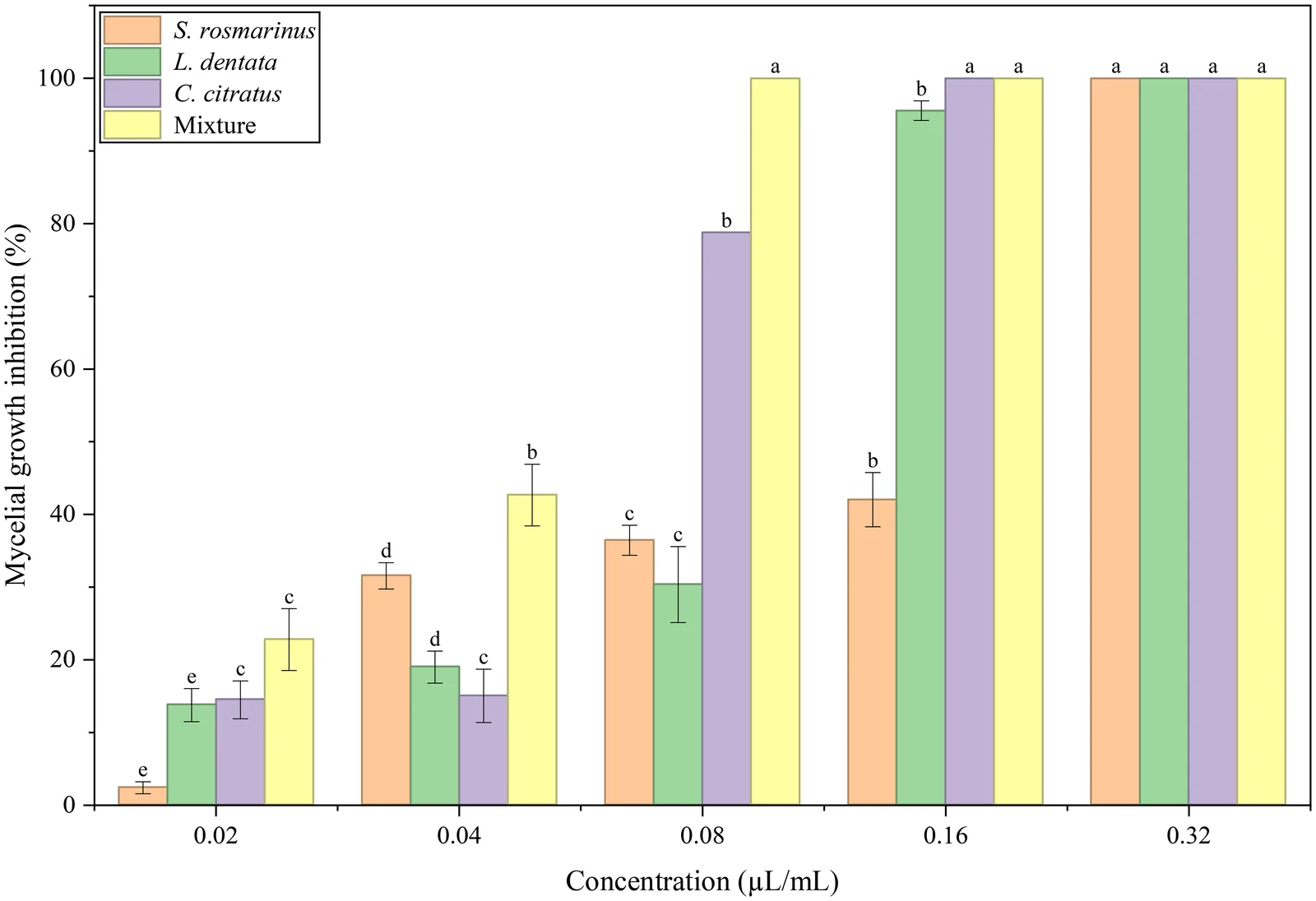
Antifungal potential of EOs and their mixture (1:1:1) on the mycelial growth of *P. digitatum*, assessed by the volatile (vapor-phase) contact assay *in vitro.* Data were presented as mean ± SD. According to the test of Kruskal-Wallis at 5%, no significant difference was found between values marked with the same letter by series.

As illustrated in **Figure 1**, mycelial growth of *P. digitatum* was significantly inhibited by all EOs and their mixture across every tested concentration (P-value< 0.001). Across all treatments, the greatest inhibitory effect was recorded at the 3.2 µL/mL air concentration, achieving complete inhibition (100%). The mixture of EOs exhibited significant effect compared to their individual application. The mycelial growth of *P. digitatum* was inhibited (100%) at the concentrations from 0.08 to 0.32 µL/mL of this mixture. At the lowest concentrations, the mixture of EOs exhibited an average of 22.80%, while *S. rosmarinus*, *L. dentata*, and *C. citratus* EOs showed lower values (2.42%, 13.78%, and 14.49%, respectively).

The results obtained for the inhibitory concentrations are shown in **Table 2**. The lowest value of IC_50_ was obtained with the mixture followed by *C. citratus* (0.0452 and 0.0648 µL/mL, respectively), while the highest values were obtained for *L. dentata* and *S. rosmarinus* (0.0994 and 0.1706 µL/mL, respectively).

**Table 2:** The inhibitory concentrations of EOs and their mixture (1:1:1) by volatile compound against the growth mycelium of P. digitatum.

Treatment	IC_50_ (µL/mL)	Standard error	95% LCL	95% UCL
*S. rosmarinus*	0.1706	0.0327	0.1034	0.2377
*L. dentata*	0.0994	0.0043	0.0906	0.1083
*C. citratus*	0.0648	0.0024	0.0599	0.0697
Mixture	0.0452	0.0016	0.0420	0.0484

(UCL and LCL: Upper and Lower Confidence Limit).

#### 3.2.2 Direct contact test

**Figure 2:**
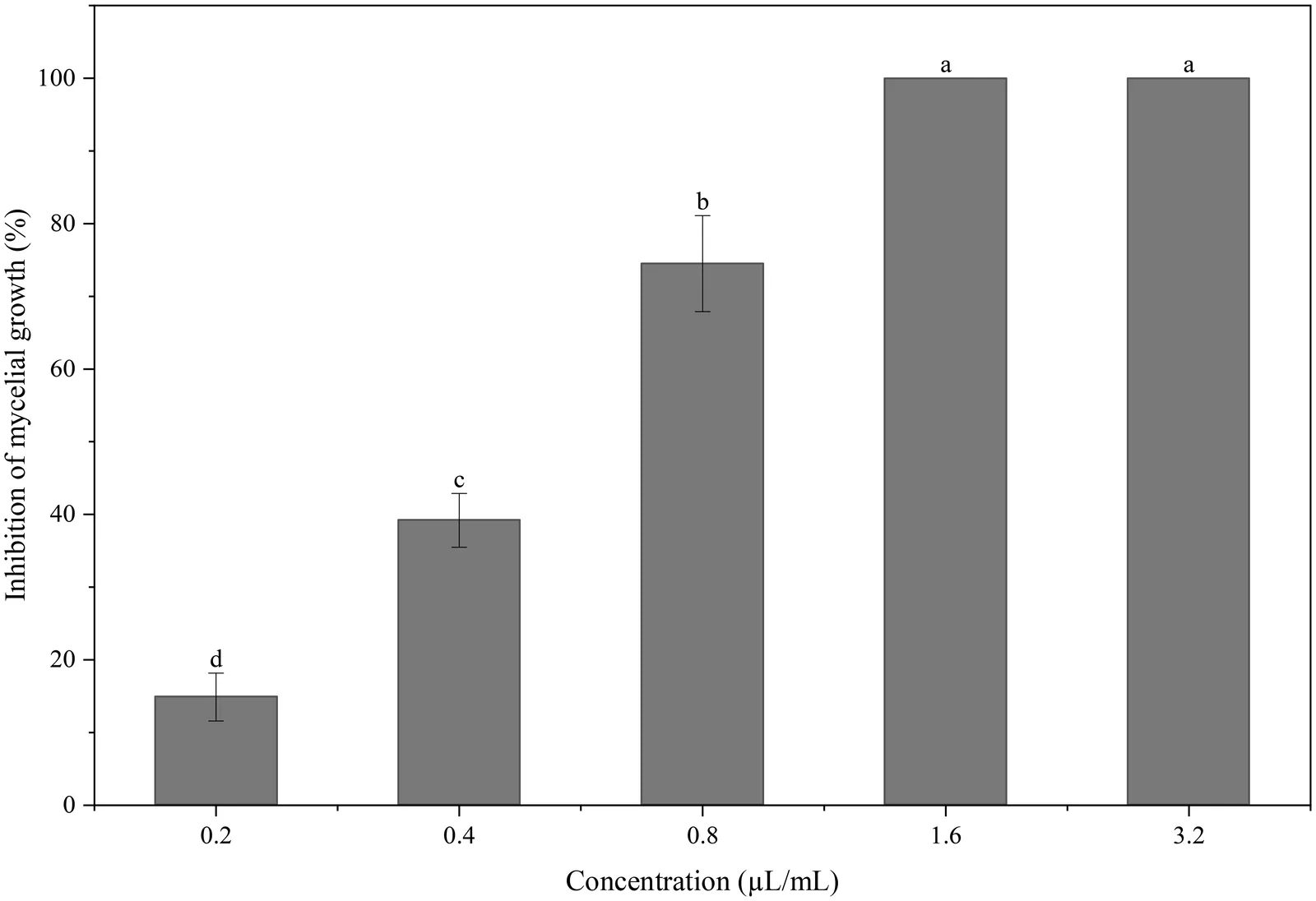
Antifungal potential of the EOs mixture on the mycelial growth of *P. digitatum*, assessed by the direct contact assay *in vitro.* Data were presented as mean ± SD. According to the test of Kruskal-Wallis at 5%, no significant difference was found between values marked with the same letter.

The results of the *in vitro* antifungal potential of the mixture of EOs on the mycelial growth of *P. digitatum* are shown in **Figure 2**. Complete inhibition (100%) was recorded at the two highest concentrations tested (1.6 and 3.2 µL/mL), whereas lower inhibition rates were observed at 0.8 and 0.4 µL/mL (74% and 39% respectively). Conversely, at 0.2 µL/mL, the inhibition rate was 14%. Moreover, the values of IC_50_ and IC_90_ obtained are 0.560 and 0.951 µL/mL (**Table 3**).

**Table 3:** The inhibitory concentrations of EOs mixture.

Inhibitory concentrations	Value (µL/mL)	Standard error	95% LCL	95% UCL
IC_50_	0.5605	0.0302	0.4985	0.6225
IC_80_	0.7826	0.0512	0.6773	0.8880
IC_90_	0.9514	0.0803	0.7863	1.1165

(UCL and LCL: Upper and Lower Confidence Limit).

#### 3.2.3 Spore germination test

**Figure 3:**
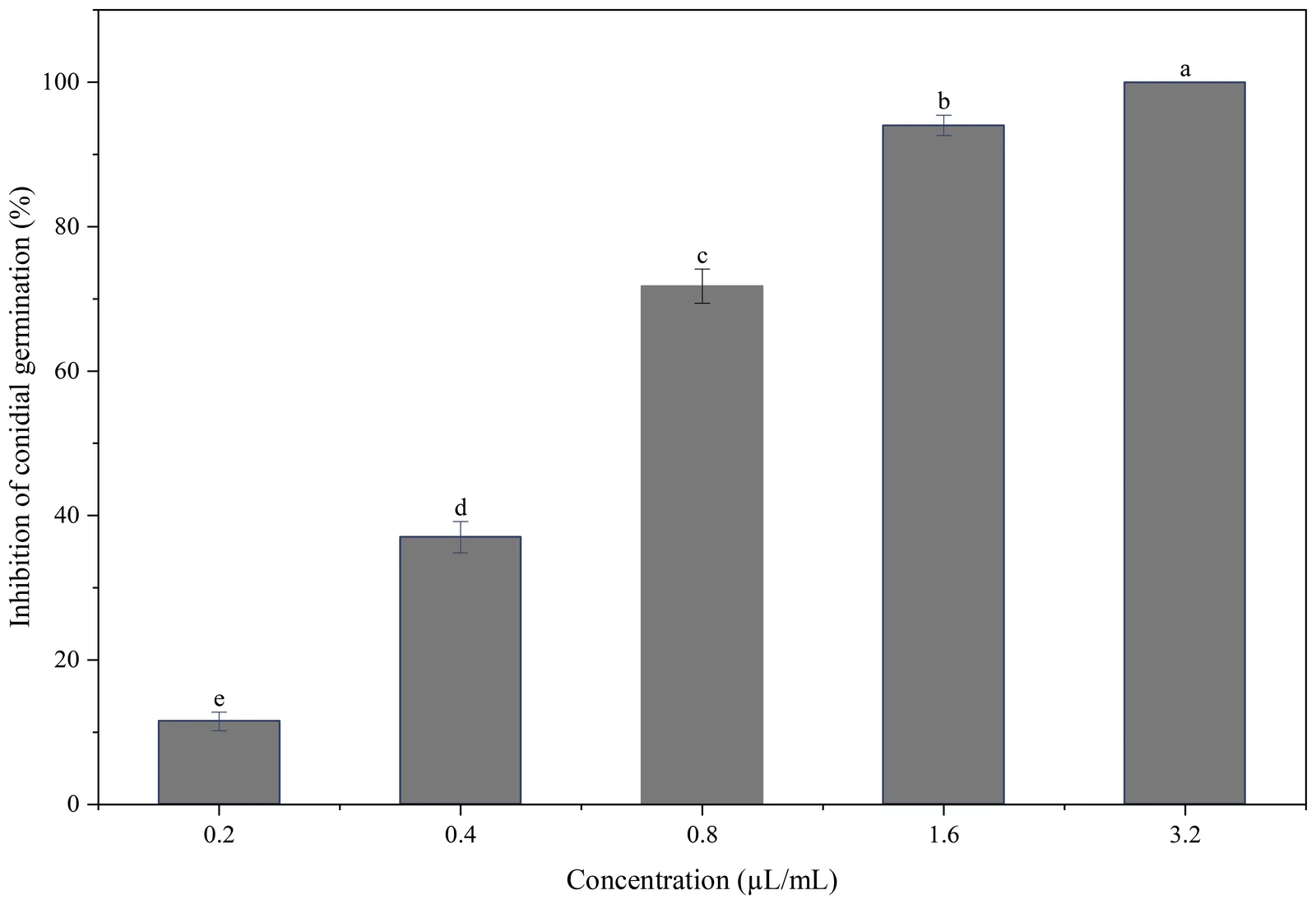
Antifungal potential of EOs mixture on the spore germination of *P. digitatum.* Data were presented as mean ± SD. According to the test of Kruskal-Wallis at 5%, no significant difference was found between values marked with the same letter.

**Figure 3** illustrates the antifungal potential of EOs mixture on spores’ germination of *P. digitatum* are shown in **Figure 3**. Complete inhibition (100%) was exhibited with 3.2 µL/mL, followed by 1.6 and 0.8 µL/mL showing rates of 94% and 71% respectively. Conversely, the lower rate (11%) was obtained for the lowest concentration. Moreover, the values of IC_50_ and IC_90_ obtained are 0.566 and 0.960 µL/mL (**Table 4**).

**Table 4:** The inhibitory concentrations values of EOs mixture against spores’ germination.

Inhibitory concentrations	Value ((µL/mL)	Standard Error	95% LCL	95% UCL
IC_50_	0.5657	0.0389	0.4831	0.6482
IC_80_	0.7899	0.0652	0.6517	0.9281
IC_90_	0.9602	0.1023	0.7434	1.1770

(UCL and LCL: Upper and Lower Confidence Limit).

##### 3.2.3.1 *In vivo* antifungal potential of EOs

**Figure 4:**
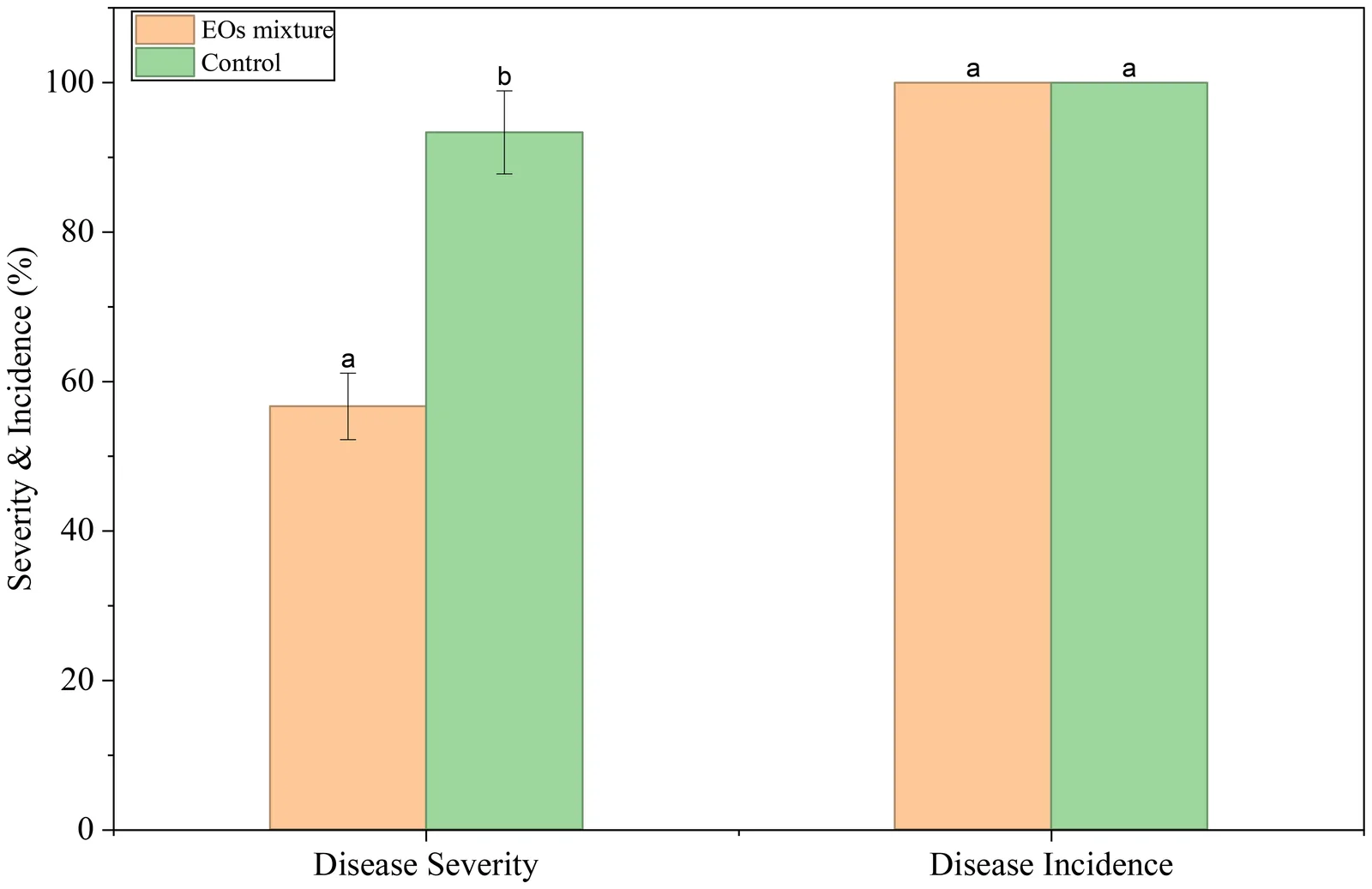
Antifungal potential of EOs mixture on the control of the green mold caused by *P. digitatum* on *C. sinensis* fruits. Data was presented as mean ± SD. According to the test of Mann-Whitney U at 5%, no significant difference was found between values marked with the same letter.

**Table 5** presents the lesion diameter and inhibition rate data obtained from the different treatments against *P. digitatum*. Lesion diameters recorded for untreated fruits reached 64.29 ± 3.95 mm and 36.05 ± 4.04 mm for treated fruits. The EO mixture at IC_90_ demonstrated antifungal activity, with an average inhibition rate of 43.92 ± 6.28%. Furthermore, mold development caused by *P. digitatum* was observed in all fruits, corresponding to a disease incidence of 100%. Nevertheless, disease severity was significantly reduced in EO-treated fruits (56.67 ± 4.44%), compared to the untreated control (93.33 ± 5.56%) (**Figure 4**).

**Table 5:** Lesion diameter and inhibition rate (%) of treatments on *C. sinensis* fruits.

Treatments	Lesion diameter (mm)	Inhibition rate (%)
Control	64.29 ± 3.95	–
EOs	36.05 ± 4.04	43.92 ± 6.28

## 4 Discussion

The chemical characterization of the essential oils (EOs) via GC-MS revealed distinct compositional profiles among the three species. The EO of *C. citratus* was found to be predominantly composed of geranial, neral—the two geometric isomers of citral—along with β-pinene. These findings corroborate with those of other studies on *C. citratus* EOs (Gharzouli et al., 2024; Jemai et al., 2025; Kayiran et al., 2025; Arafa et al., 2026). *S. rosmarinus* EO was found to be rich in 1,8-cineol, camphor, and α-pinene. This profile is consistent with the findings of previous studies on *S. rosmarinus*, which have reported camphor, α-pinene, and 1,8-cineole as the dominant compounds. In a similar manner, the EO of *L. dentata* was found to contain high levels of camphor, 1,8-cineole, and β-pinene, a finding that is in accordance with previous reports on the EO of *L. dentata*, which have consistently identified camphor, 1,8-cineole, and β-pinene as the predominant compounds, with camphor typically representing the most abundant constituent (Jemai et al., 2025; Khan et al., 2025; Hamdeni et al., 2026; Vetere et al., 2026).

All EOs exhibited *in vitro* antifungal activity against *P. digitatum* in the volatile assay, with inhibition of mycelial growth increasing in a dose-dependent manner. Among the individual EOs tested, *C. citratus* EO was found to be the most effective, a result that can be attributed directly to its extreme citral content. This lipophilic monoterpene has been proven antifungal properties against *P. digitatum* through a variety of mechanisms, including the permeabilization of the cell membrane, the leakage of cellular components, the disruption of cell integrity, and ultimately cell death (Tao et al., 2014; Saraf et al., 2024; Wang et al., 2024; Hu et al., 2025). Furthermore, among the mechanisms proposed to explain citral’s antifungal potential against *P. digitatum*, interference with oxidative phosphorylation has been highlighted as particularly significant. This mechanism severely impacts mitochondrial complexes (Zheng et al., 2015; OuYang et al., 2018). Additionally, the mechanism of action of citral is further explained by its inhibitory effect on ergosterol biosynthesis. Depletion of ergosterol from the membrane leads to accumulation of lanosterol, its precursor, and disruption of membrane structural integrity (OuYang et al., 2016). Furthermore, the EOs of *S. rosmarinus* and *L. dentata* exhibited significant antifungal activity. The antifungal efficacy of these EOs is frequently associated with the individual or synergistic effects of their major and minor components. However, the predominance of a given compound does not necessarily imply a greater contribution to the observed activity, as minor constituents may exhibit higher intrinsic bioactivity despite their lower concentration. It has been reported that 1,8-cineole inhibits fungal growth by enhancing cell membrane permeability. Meanwhile, camphor contributes to cytoplasmic leakage and metabolic disruption (Moo et al., 2021; Kong et al., 2022).

The co-occurrence of bioactive compounds in *L. dentata*, *S. rosmarinus* and *C. citratus* EOs, gives rise to a chemically diverse mixture comprising complementary functional groups, including oxygenated monoterpenes, and monoterpene hydrocarbons. This complex composition has the potential to synergistically target multiple fungal cellular structures. The mixture of the three EOs showed significantly greater inhibition of *P. digitatum* mycelial growth in the volatile phase compared to each individual oil. This result is in line with the growing body of literature on the synergistic potential of EO combinations (Regnier et al., 2014; Ji et al., 2019; Chafiki et al., 2025; Khaled et al., 2025). The search for synergistic effects through EO mixtures has been a major research focus in postharvest disease management, with mixture design approaches consistently revealing enhanced antifungal efficacy against *P. digitatum* compared to individual oils. In a closely comparable study, Khaled et al. (2025) evaluated mixtures of *S. rosmarinus*, *Origanum vulgare*, and *Mentha rotundifolia* EOs against *P. digitatum*, finding that the optimized mixture exhibited significantly greater inhibitory activity than any of the individual components, confirming the synergistic interaction among the oil constituents. The synergistic effect observed in our study can be mechanistically explained by the chemical complementarity of the mixture. Similarly, Chafiki et al. (2025) assessed mixtures of *S. rosmarinus*, *L. dentata* and *C. citratus* against *B. cinerea*, finding that the mixture at ratio (1:1:1) exhibited significantly greater inhibitory activity than their individual application. Furthermore, the inhibition of mycelial growth and spore germination of *P. digitatum* was confirmed by the incorporation method. Similarly, da Silva et al. (2025) showed that the mixture of major constituents of the cinnamon bark EO (cinnamaldehyde, o-methoxy cinnamaldehyde, and cinnamyl acetate) and oregano EO (carvacrol, ρ-cymene, and thymol) completely inhibited the mycelial growth of *P. digitatum* at a concentration of 0.50 μL/mL, while the combination of the major constituents of the rosemary pepper EO (thymol, ρ-cymene, and caryophyllene) exhibited complete inhibition of the pathogens in the MIC of MIC of 0.75 μL./mL.

*In vivo* experiments proved that treatment of orange fruits with EOs mixture resulted in the inhibition of green mold development caused by *P. digitatum* compared to the untreated control. This result confirms that the mixture retains significant biocontrol activity under realistic conditions, despite the biological complexity of the fruit surface environment. In a directly comparable approach, Moussa et al. (2021) showed that a 0.5 mg/mL solution of thymol decreased the surface rot by 80.0% in oranges inoculated with *P. digitatum*. The *in vivo* inhibition of the mixture may be further improved by optimizing the delivery system. Microencapsulation, nano emulsion formulation, or incorporation into edible coatings could protect the volatile compounds and extend their contact time with the fruit surface, as demonstrated in several recent studies. As well as Shaukat et al. (2026) showed that oregano EO nanoemulsion coatings applied to citrus fruits showed significantly enhanced antifungal efficacy against *P. digitatum* compared to unformulated EO, while maintaining fruit quality parameters throughout cold storage.

The results of this study place the EOs mixture from the three plants studied among natural compounds showing promising fungicidal activity currently registered for green mold control in citrus. Likewise, EOs are generally recognized as safe, biodegradable, and consistent with consumer demand for clean-label and sustainable food preservation strategies. The activity of EO mixture against *P. digitatum* opens avenues for their use in integrated postharvest disease management programs, potentially in combination with physical treatments, biological agents, or low-residue conventional fungicides. Moreover, while a multi-target mechanism of action has been proposed for EOs mixtures in literature, this remains a hypothesis in the absence of direct mechanistic evidence in the present study. Further investigations, including bioassay-guided fractionation and mechanistic assays, would be needed to clarify the specific contribution and mode of action of individual components.

Furthermore, Synthetic postharvest fungicides like Imazalil and Thiabendazole provide a high industry baseline for green and blue mold control (Kinay et al., 2007), however, growing regulatory restrictions and consumer demand drive the need for natural alternatives. In this study, the inclusion of an emulsifier control (Tween-80) validated that the observed disease suppression was driven specifically by essential oil bio-efficacy. Contextualizing these findings against reported literature benchmarks for standard chemical treatments confirms that optimized essential oil formulations offer a promising bio-based framework for citrus decay reduction.

## 5 Conclusion

This study highlights the promising antifungal potential of three EOs derived from *S. rosmarinus*, *L. dentata*, and *C. citratus*, and their mixture, against *P. digitatum*. Notably, combining the EOs significantly enhanced antifungal efficacy compared to their individual use. Such combinations offer a way to preserve biodiversity, reduce reliance on synthetic chemical inputs, and mitigate the environmental and health risks associated with conventional pesticides. Future research should focus on optimization, formulations, evaluating their stability, and assessing the cost-effectiveness of EO-based treatments under field conditions, in order to support their broader adoption in agroecological and organic farming systems.

## Data Availability

The original contributions presented in the study are included in the article/supplementary material. Further inquiries can be directed to the corresponding author.
